# Impact of overweightness and critical weight loss on overall survival in patients with hepatocellular carcinoma initially treated with chemoembolization

**DOI:** 10.1093/gastro/goz040

**Published:** 2019-08-28

**Authors:** Zhen-Xin Chen, Zhi-Wei Jian, Xi-Wen Wu, Jun-Cheng Wang, Jing-Yuan Peng, Chun-Yu Huang, Xiang-Ming Lao

**Affiliations:** 1 Department of Hepatobiliary and Pancreatic Surgery, Sun Yat-sen University Cancer Center, State Key Laboratory of Southern China, Collaborative Innovation Center for Cancer Medicine, Guangzhou, Guangdong, P. R. China; 2 Department of Endoscopy, Sun Yat-sen University Cancer Center, State Key Laboratory of Southern China, Collaborative Innovation Center for Cancer Medicine, Guangzhou, Guangdong, P. R. China

**Keywords:** overweightness, critical weight loss, hepatocellular carcinoma, chemoembolization, overall survival

## Abstract

**Background:**

The effects of overweightness and weight loss on the development and prognosis of hepatocellular carcinoma (HCC) remain unclear. In this study, we aimed to evaluate the impact of overweightness and weight loss on the survival of patients with intermediate/advanced HCC receiving chemoembolization as initial treatment.

**Methods:**

We examined 1,170 patients who underwent chemoembolization as initial treatment for Barcelona-Clínic Liver Cancer stages B and C HCC at Sun Yat-sen University Cancer Center (Guangzhou, China) between December 2009 and May 2015. A baseline body mass index (BMI) of ≥23 kg/m^2^ was defined as overweight, and body-weight loss of ≥5.0% from baseline was defined as critical weight loss (CWL). Cox regression analysis was used to determine the association between overweightness or CWL and overall survival (OS).

**Results:**

The median survival time was 16.8 (95% confidence interval, 13.9–19.7) months and 11.1 (95% confidence interval, 10.0–12.2) months in the overweight and non-overweight groups (log-rank test, *P *<* *0.001), respectively. Cox multivariate analysis identified overweightness as an independent protective prognostic factor for OS (*P *<* *0.001). Subgroup stratification analysis revealed a significant association between overweightness and survival among patients receiving further treatment (*P *=* *0.005), but not in those not receiving further treatment (*P *=* *0.683). Multivariate analysis showed that both overweightness and CWL were independent prognostic factors for OS among patients receiving further treatment.

**Conclusion:**

Among patients with intermediate- or advanced-stage HCC initially treated with chemoembolization, overweightness was associated with longer OS. Furthermore, CWL was an independent adverse prognostic factor for OS in patients receiving additional treatment.

## Introduction

Hepatocellular carcinoma (HCC) is the sixth most common cancer and the second leading cause of cancer mortality worldwide [1]. The incidence of HCC varies widely between geographic regions and is the highest in the Asia-Pacific region, where HCC is one of the leading public-health concerns [2].

Studies have shown that overweightness is associated with the risk of cancers, such as HCC, and mortality [3, 4]. However, data regarding the association between overweightness and overall survival (OS) of patients undergoing curative resection for early-stage HCC are sporadic, and the results are controversial. Some studies have demonstrated that overweightness is a favorable factor for the OS of patients after hepatic resection [5], whereas other studies indicate that overweightness has either no effect or a negative effect on the OS [6–9]. Moreover, to the best of our knowledge, there are hardly any reports regarding the relationship between survival and overweightness in patients with HCC of intermediate-stages, such as Barcelona Clinic Liver Cancer (BCLC) stages B and C, who receive transcatheter arterial chemoembolization (TACE) as a standard therapy [10–12]. Only one study has specifically evaluated the impact of obesity on the short-term (1 month–2 months) radiologic outcomes in patients with HCC treated with TACE, and no data are available regarding the survival time [13].

Moreover, weight loss during anticancer treatment has been reported to be a negative prognostic factor for the OS of patients with various types of tumor, other than HCC [14–16]. Nevertheless, the impact of weight loss after initial TACE on the prognosis of HCC remains unclear.

In the present study, we aimed to evaluate the long-term effect of overweightness and weight loss on the OS of patients with HCC of BCLC stage B or C after initial TACE in a large cohort.

## Patients and methods

### Patients and inclusion criteria

We reviewed the medical records of 1,370 consecutive patients who were diagnosed with HCC and received initial TACE in the Department of Hepatobiliary Oncology of Sun Yat-sen University Cancer Center (Guangzhou, China) between December 2009 and May 2015. This retrospective study was approved by the Institutional Review Board at Sun Yat-sen University Cancer Center.

Baseline examinations were performed in the week prior to TACE and included the following parameters: serum liver-function tests (alanine aminotransferase [ALT], aspartate aminotransferase [AST], alkaline phosphatase, total bilirubin [TB], and albumin [ALB]); creatinine levels; prothrombin time (PT); activated partial thromboplastin time (APTT); alpha-fetoprotein (AFP) levels; complete blood counts; serum HBV DNA quantification; detection of hepatitis B surface antigen (HBsAg), hepatitis B surface antibody (HBsAb), hepatitis B core antibody (HBcAb), hepatitis B e antigen (HBeAg), hepatitis B e antibody (HBeAb), and anti-hepatitis C virus (HCV) antibody (HCV Ab); and chest radiography. The diagnosis of HCC was based on the criteria established by the European Association for the Study of the Liver [17]. Tumor characteristics and BCLC stage were evaluated through imaging and/or intra-operative observation.

The inclusion criteria were as follows: HCC patients that were initially treated with TACE, Child-Pugh class A or B liver disease, HCC of BCLC stage B or C, adequate renal function (serum creatinine <124 μmol/L), and good tolerance of TACE. Exclusion criteria were as follows: extra-hepatic tumor metastasis, history of prior anticancer treatment for HCC, Child-Pugh grade C liver function, any other malignancy, concurrent non-malignant severe illness(es), missing weight-measurement data, or other relevant clinical information at baseline. A final total of 1,170 patients were enrolled in this study.

### Measurement and grouping

The patients’ baseline height and body weight were recorded in the week prior to TACE. The BMI of individuals was calculated as their weight (kg) divided by their height (m) squared and was categorized according to the Western Pacific Regional Office of WHO (WPRO) recommendations for the Asian population: underweight (<18.5 kg/m^2^), normal weight (18.5 kg/m^2^–22.9 kg/m^2^), overweight at-risk (23.0 kg/m^2^–24.9 kg/m^2^), and obese (≥25.0 kg/m^2^) [18]. With respect to the OS, no difference was found between the overweight at-risk (23.0 kg/m^2^–24.9 kg/m^2^) and obese (≥25.0 kg/m^2^) sub-categories. Furthermore, no difference was noted in the OS of underweight (<18.5 kg/m^2^) and normoweight (18.5 kg/m^2^–22.9 kg/m^2^) sub-categories (Supplementary Figure 1). Therefore, we considered both overweight at-risk and obese sub-categories together as the overweight group (≥23.0 kg/m^2^), and the underweight and normal-range sub-categories as the non-overweight group (<23.0 kg/m^2^). A similar method of grouping (overweight and non-overweight groups) has been widely used in other studies [5, 19]. The differences in the outcomes of the overweight and non-overweight groups were compared. For patients who required post-TACE treatment that necessitated re-hospitalization, the post-TACE weight measurements were available (*n *=* *654), and the weight loss and weight gain could be calculated. The baseline and post-TACE body-weight measurements were designated as W0 and W1, respectively. The extent of weight change was calculated as a percentage using the following formula: (W1 − W0)/W0 × 100%. For patients that did not receive subsequent treatment, the post-TACE weight measurements were unavailable, and the weight changes after initial TACE could not be calculated in the present study. We evaluated the follow-up serum ALB of HCC patients 1 month after TACE treatment (follow-up serum ALB data were available in 893 patients). Levels lower than 35 g/L of serum albumin were indicated as malnutrition as previously reported [20–22].

## Treatments

### TACE procedure

TACE was performed as described previously [23, 24]. After the catheter tip was advanced into the tumor-feeding arteries, one or several chemotherapeutic agents mixed with lipiodol were slowly injected. Gelatin sponge particles were injected in some sessions if the territory corresponding to the chemoembolized artery did not show stagnancy of blood flow. The chemotherapeutic agents administered included epirubicin (60 mg/session) and/or mitomycin (6 mg/session), carboplatin (300 mg/session), lobaplatin (50 mg/session), and floxuridine (500 mg/session). The choice of the combinations of anticancer agents, lipiodol emulsion dosage, and gelform applied was made on a case-by-case basis. The cycles of TACE were recorded.

### Subsequent treatment

After the initial TACE, the patients were followed up and received subsequent treatment as and when appropriate, including repeated TACE, local ablation, hepatectomy, or Sorafenib therapy, on a case-by-case basis. The subsequent treatment choices were based on tumor burden, liver function, and the patient’s request. Specifically, hepatic resection was performed on patients whose tumor shrank and the gross residual lesion could potentially be resected. Local ablation (including radiofrequency ablation and microwave ablation) was offered to patients whose residual lesion was ≤3.0 cm and the procedure could potentially eliminate all gross lesions shown radiologically, usually when embolization was technically inaccessible. Repeat TACE at 6-week to 8-week intervals was offered to patients whose residual tumor enhancement and residual tumor vascularity could be seen on CT imaging or hepatic artery angiographs without contraindications to the repeat TACE. Contraindications to repeat TACE included (i) Eastern Collaborative Oncology Group (ECOG) score >2, (ii) deterioration of liver function to Child-Pugh C, (iii) severe extra-hepatic disease, (iv) portal-vein tumor thrombus with complete vessel obstruction, (v) technically inaccessible embolization (exclusive supply of the residual tumors by extra-hepatic collateral arteries, the catheter not able to reach the target hepatic artery, or obstruction of the tumor-feeding artery), and (vi) refusal for the subsequent TACE procedure. For patients with tumor progression without contraindications to TACE, repeat TACE combined with Sorafenib treatment was recommended. Patients who were not appropriate for any of the subsequent treatments above were recommended to receive Sorafenib. Conservative treatments were applied to patients with terminal HCC or ECOG score >2. Liver transplantation was not available in our institute, and none of the patients enrolled in the current study were offered to undergo liver transplantation. The follow-up period ended on 8 June 2018.

#### Statistical analysis

The demographic data (mean, standard deviation, and percentage) were collected and calculated. The analyses were conducted using the independent Student’s *t*-test, ANOVA, and Chi-square test, as appropriate. Logistic-regression analysis was performed to identify the possible independent factors associated with the maintenance/improvement of nutritional status. Kaplan–Meier methods and log-rank tests were used for survival analysis. The OS was calculated from the date of initiation of TACE to the date of death or the last follow-up. The Cox proportional-hazards model was used for the univariate survival analysis to determine the association between the individual clinical variables and the OS. All variables with *P* < 0.1, in addition to age and sex, were subsequently subjected to multivariate Cox regression to determine the hazards ratios (HRs) and the independence of effects. The proportional-hazards assumption was checked by the graphic inspection of the linearity of the hazards over time, using log–log plots, and plotting Schoenfeld residuals over time. A *P*-value <0.05 was considered statistically significant. All statistical tests were two-sided. Data were analysed using the SPSS 18.0 software (SPSS, Inc., Chicago, IL, USA).

## Results

### Study population and clinical features

The clinical features of the 1,170 patients who met the inclusion criteria are displayed in Table 1. The patients included 1,061 men and 109 women, with a median age of 51.7 years. Further, 723 patients (61.7%) had a BMI of <23.0 kg/m^2^ (forming the non-overweight group) and 447 (38.3%) patients had a BMI of ≥23.0 kg/m^2^ (forming the overweight group). In total, 504 patients received only a single cycle of TACE, without any additional treatment, whereas the remaining 666 patients received post-TACE treatment, including surgery, further TACE cycles, ablation, or Sorafenib treatment. In all, 174, 156, and 66 patients underwent surgery, ablation, and Sorafenib treatment after initial TACE, respectively. The proportion of patients who received post-TACE treatment was significantly higher in the overweight group (63.5%, 284/447) than in the non-overweight group (52.8%, 382/723; *P *<* *0.001). In addition, no intergroup differences were noted in terms of the classification of serum ALT, ALB, TB, PT, and AFP levels; Child-Pugh score; status of viral infection; and BCLC stage (Table 1).

**Table 1.** Characteristics of all included patients grouped by body mass index

Parameter	Total (*n *=* *1,170)	BMI <23 kg/m^2^ (*n *=* *723)	BMI ≥23 kg/m^2^ (*n *=* *447)	*P*-value^b^
Age (years)	51.7 ± 11.7	51.5 ± 12.1	51.9 ± 11.0	0.546
Sex (female vs male)	109: 1,061	74: 649	35: 412	0.203
Diabetes (no vs yes)	1,060: 110	671: 52	389: 58	*0.001*
Treatments for diabetes (no vs yes)	1,113: 57	693: 30	420: 27	0.144
WBC (× 10^9^/L)	6.85 ± 2.37	6.74 ± 2.29	7.02 ± 2.48	0.051
HGB (g/L)	138.85 ± 19.74	137.15 ± 20.54	141.59 ± 18.07	*<0.001*
PLT (× 10^9^/L)	196.27 ± 90.79	201.23 ± 92.33	188.24 ± 87.76	*0.016*
ALT (U/L)	59.30 ± 44.40	58.67 ± 40.88	60.32 ± 49.61	0.554
AST (U/L)	74.72 ± 54.76	78.03 ± 55.99	69.35 ± 52.34	*0.007*
ALB (U/L)	39.75 ± 4.81	39.57 ± 5.02	40.06 ± 4.44	0.082
TBIL (μmol/L)	16.80 ± 9.39	16.56 ± 8.16	17.19 ± 11.09	0.300
PT (s)	12.30 ± 1.40	12.28 ± 1.24	12.34 ± 1.63	0.461
APTT (s)	27.98 ± 4.49	28.21 ± 4.57	27.62 ± 4.35	*0.028*
AFP (≤25 vs >25 ng/mL)	301:869	179:544	122:325	0.371
BCLC_stage (B vs C)	772:398	474:249	298:149	0.745
Child_Pugh_score (A vs B)	1143:27	705:18	438:9	0.744
Viral_infection (no vs HBV+HCV vs HCV vs HBV)	59:22:6:1,083	34:14:3:672	25:8:3:411	0.838
Subsequent_therapy^a^ (yes vs no)	666:504	382:341	284:163	*<0.001*
Cycles of TACE (one vs more than one)	625:545	412:311	213:234	*0.002*
Resection after TACE (yes vs no)	174:996	99:624	75:372	0.150
Local ablation after TACE (yes vs no)	156:1,014	69:654	87:360	*<0.001*
Sorafenib therapy after TACE (yes vs no)	66:1,104	33:690	33:414	0.095

a Subsequent therapy means any treatment after TACE, including surgery, local ablation, or Sorafenib therapy.

b Differences between patients with body mass index (BMI) ≥23 and those with BMI <23. WBC, white blood cells; HGB, hemoglobin; PLT, platelets; ALT, alanine aminotransferase; AST, aspartate aminotransferase; ALB, serum albumin; TBIL, total bilirubin; PT, prothrombin time; APTT, activated partial thromboplastin time; AFP, α-fetoprotein; BCLC, Barcelona Clinic Liver Cancer; HBV, hepatitis B virus; HCV, hepatitis C virus; TACE, transcatheter arterial chemoembolization.

### Univariable and multivariable analysis of factors related to survival in all patients

**Figure 1.**
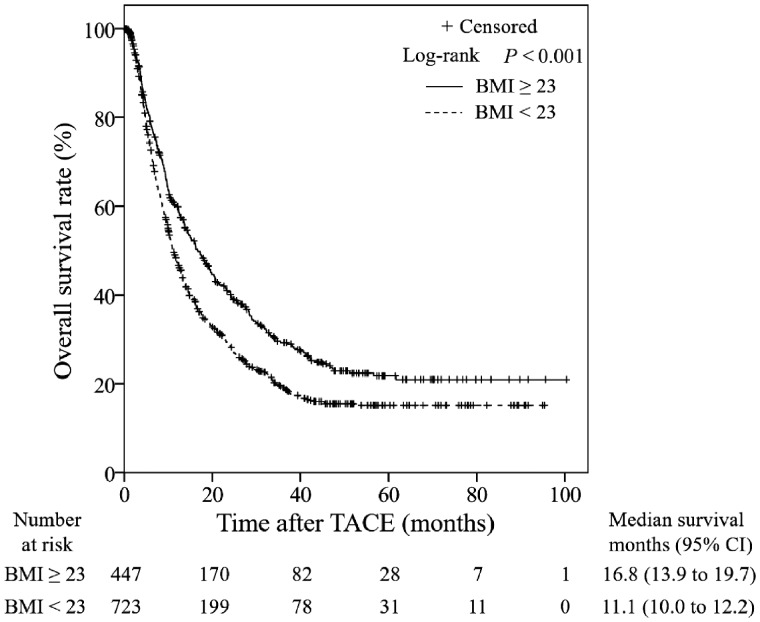
Kaplan–Meier curves of overall survival (OS) for 1,170 hepatocellular carcinoma (HCC) patients by body mass index (BMI) level. Overweight patients have significantly higher OS than non-overweight patients. TACE, transarterial chemoembolization; CI, confidence interval.

The median follow-up period for all patients was 10.8 months. The median survival time (MST) was 12.9 months (95% confidence interval [CI], 11.7–14.1 months) in all patients. The MST in the overweight group was 16.8 months (95% CI, 13.9–19.7 months), which was significantly longer than that of the non-overweight group (MST, 11.1 months; 95% CI, 10.0–12.2 months; *P *<* *0.001; Figure 1). Univariate analysis revealed that PT (>13.5 s), APTT (≤34 s), AST (≤40 U/L), ALT (≤40 U/L), ALB (>40 g/L), TB (≤20.5 μmol/L), AFP (≤25 ng/mL), BCLC stage (B stage), and BMI (≥23 ) were significant factors related to prolonged OS (Table 2). Multivariate analysis identified that the BMI was an independent prognostic factor for the OS (*P *<* *0.001), together with AST, ALB, TB, AFP, and BCLC stage (Table 2).

**Table 2.** Univariate and multivariate analysis of factors related to survival in all HCC patients initially treated with transcatheter arterial chemoembolization

Variable	Univariate analysis *P*-value	Multivariate analysis		
		Hazard ratio	95% CI	*P*-value
Age (≤45 vs >45 years)	0.127	0.929	0.799–1.080	0.339
Sex (female vs male)	0.778	1.092	0.867–1.376	0.454
PT (≤13.5 vs >13.5 s)	0.004	0.970	0.776–1.213	0.791
APTT (≤34 vs >34 s)	0.022	1.103	0.852–1.429	0.457
AST (≤40 vs >40 U/L)	<0.001	1.345	1.108–1.633	*0.003*
ALT (≤40 vs >40 U/L)	0.007	1.070	0.913–1.253	0.403
ALB (≤40 vs >40 U/L)	<0.001	0.797	0.689–0.921	*0.002*
TB (≤20.5 vs >20.5 μmol/L)	<0.001	1.192	1.006–1.413	*0.043*
AFP (≤25 vs >25 ng/mL)	<0.001	1.354	1.150–1.594	*<0.001*
BCLC_stage (B vs C)	<0.001	1.847	1.595–2.139	*<0.001*
BMI (<23 vs ≥23 kg/m^2^)	<0.001	0.776	0.673–0.895	*<0.001*

CI, confidence interval. Other abbreviations as in Table 1.

### Stratified analysis based on the BCLC staging

#### BCLC B-stage subgroup

**Figure 2.**
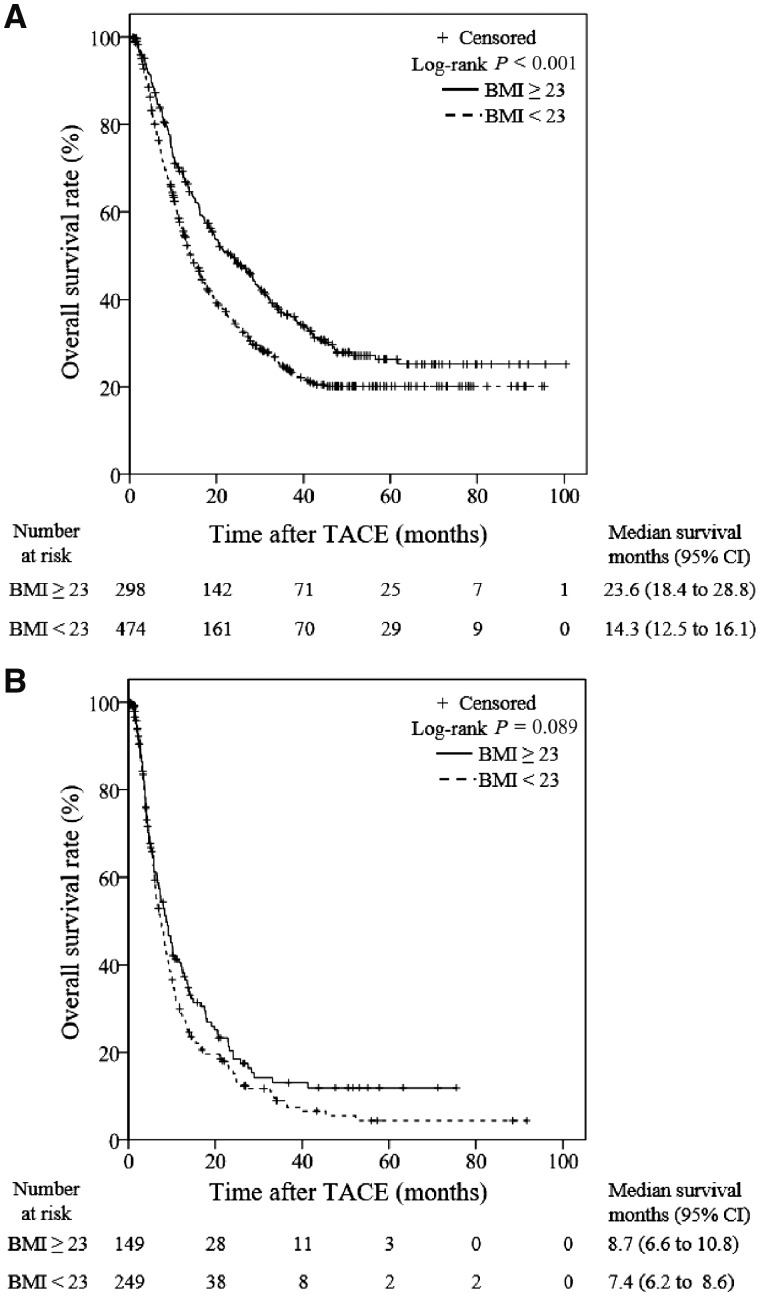
Kaplan–Meier subgroup analysis stratified according to BCLC stage. (A) For the 772 patients with BCLC B-stage HCC, the overall survival (OS) rates of overweight patients are significantly higher than those of non-overweight patients. (B) For the 398 patients with BCLC C-stage HCC, there is no significant difference in OS between overweight and non-overweight patients. TACE, transarterial chemoembolization; CI, confidence interval.

There were 772 patients with BCLC stage-B disease. The MST in the overweight group was significantly longer (23.6 months; 95% CI, 18.4–28.8 months) than that in the non-overweight group (14.3 months; 95% CI, 12.5–16.1 months; Figure 2A). Univariate analysis identified that the following factors were significantly associated with the OS for patients with BCLC B-stage disease: diabetes; PT; APTT; serum levels of AST, ALT, ALB, TB, and AFP; and BMI. Multivariate analysis revealed that the BMI was an independent risk factor for the prognosis of HCC patients with BCLC stage-B disease (*P *=* *0.001), along with APTT and serum AST, ALB, TB, and AFP levels (Table 3).

**Table 3.** Univariate and multivariate analysis of factors related to survival in HCC patients with Barcelona Clinic Liver Cancer stage-B disease

Variable	Univariate analysis *P*-value	Multivariate analysis		
		Hazard ratio	95% CI	*P*-value
Age (≤45 vs >45 years)	0.626	1.022	0.840–1.244	0.824
Sex (female: male)	0.167	1.223	0.931–1.606	0.148
Diabetes (no vs yes)	0.062	0.855	0.631–1.159	0.312
PT (≤13.5 vs >13.5 s)	0.011	1.079	0.795–1.464	0.625
APTT (≤34 vs >34 s)	0.002	1.379	1.002–1.898	*0.049*
AST (≤40 vs >40 U/L)	<0.001	1.441	1.147–1.811	*0.002*
ALT (≤40 vs >40 U/L)	0.043	1.020	0.835–1.247	0.845
ALB (≤40 vs >40 U/L)	<0.001	0.825	0.690–0.987	*0.036*
TB (≤20.5 vs >20.5 μmol/L)	<0.001	1.387	1.113–1.728	*0.004*
AFP (≤25 vs >25 ng/mL)	<0.001	1.382	1.138–1.679	*0.001*
BMI (<23 vs ≥23 kg/m^2^)	<0.001	0.738	0.616–0.885	*0.001*

CI, confidence interval. Other abbreviations as in Table 1.

#### BCLC C-stage subgroup

In total, 398 patients had BCLC C-stage diseases. The MST was 8.7 months (95% CI, 6.6–10.8 months) in the overweight subgroup and 7.4 months (95% CI, 6.2–8.6 months) in the non-overweight subgroup. The Kaplan–Meier analysis revealed that there was no significant difference in the OS between the overweight subgroup and the non-overweight subgroup among the patients with BCLC stage-C disease (*P *=* *0.089; Figure 2B).

### Stratified analysis based on the presence or absence of post-TACE subsequent treatment

The proportion of patients receiving subsequent treatment after initial TACE was unevenly distributed between the two groups. Subsequent treatment affected the OS. The MST in patients who received subsequent treatment (20.6 months; 95% CI, 18.0–23.2 months) was significantly longer than that in patients who did not receive additional treatment (6.6 months; 95% CI, 5.9–7.3 months; Supplementary Figure 2). Stratified analysis was performed according to whether post-TACE treatment was administered.

#### Subgroups with no subsequent treatment

**Figure 3.**
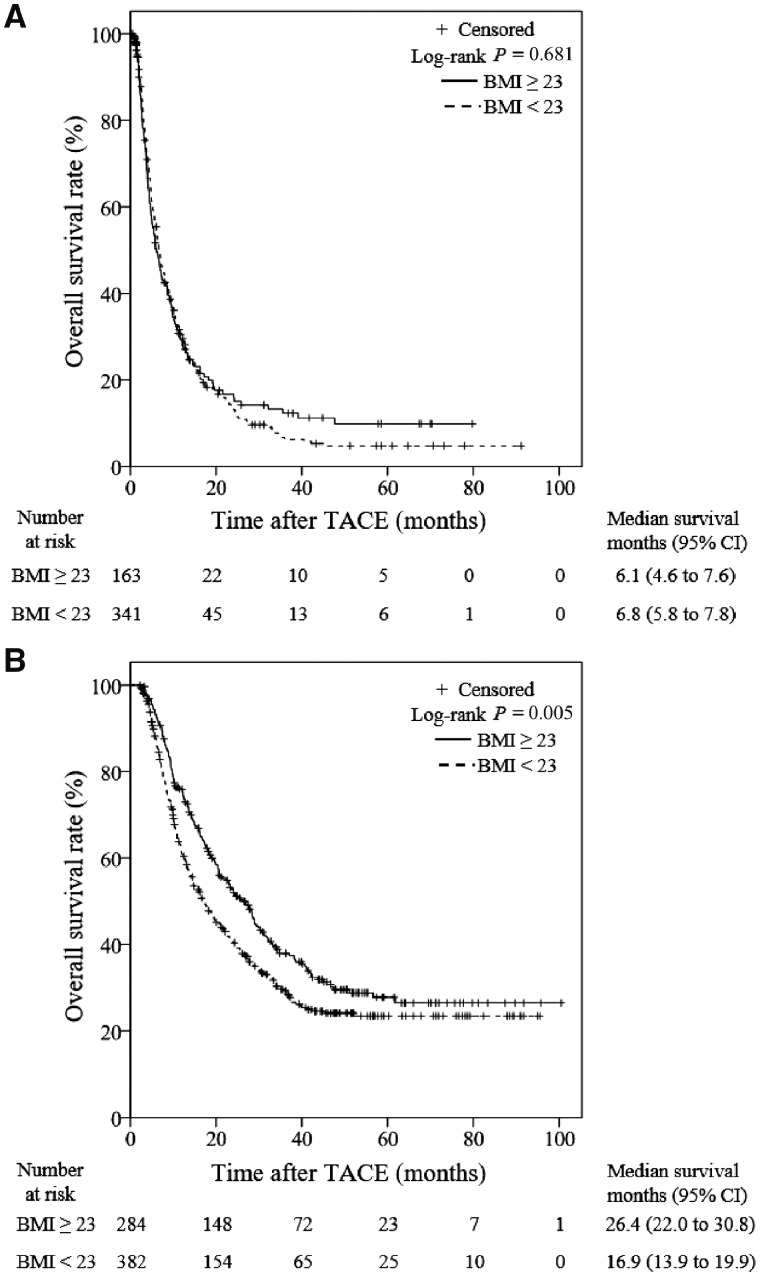
Kaplan–Meier subgroup analysis stratified according to whether subsequent treatment was administered after transarterial chemoembolization (TACE). (A) For the 504 patients without further treatment, no significant difference is noted between the overweight and non-overweight patients. (B) For the 666 patients who received further treatment, the OS rates of overweight patients are significantly higher than those of non-overweight patients. TACE, transarterial chemoembolization; CI, confidence interval.

Kaplan–Meier analysis revealed that there was no significant difference in the OS between the overweight group and the non-overweight group among patients who did not receive post-TACE treatment (6.1 [95% CI, 4.6–7.6] months vs 6.8 [95% CI, 5.8–7.8] months, *P *=* *0.681; Figure 3A).

#### Subgroups with subsequent treatment

**Figure 4.**
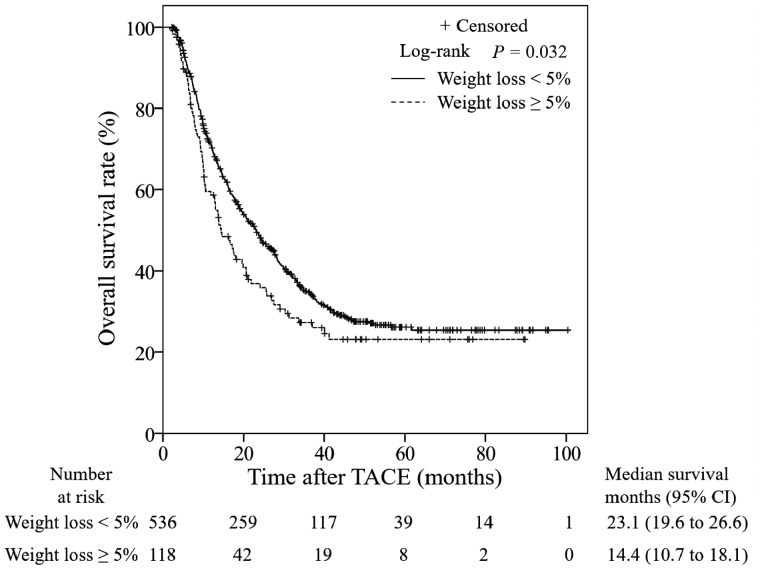
Kaplan–Meier curves of overall survival (OS) for 654 hepatocellular carcinoma (HCC) patients receiving further treatment after transarterial chemoembolization (TACE), classified by weight-loss status. The OS rates of the group with weight loss <5% are significantly greater than those of the group with weight loss ≥5%. TACE, transarterial chemoembolization; CI, confidence interval.

In total, 666 patients received post-TACE treatment. Except for 12 patients whose weight measurements following the initial chemoembolization were missing, the post-TACE weight measurements of 654 patients were recorded before the subsequent treatment. Thus, the changes in weight could be calculated in these patients. Among the patients who received post-TACE treatment, the MST for the overweight group (26.4 months; 95% CI, 22.0–30.8 months) was significantly longer than that in the non-overweight group (16.9 months; 95% CI, 13.9–19.9 months; Figure 3B). Furthermore, Kaplan–Meier analysis identified that the OS was longer among patients with weight loss <5% than among patients with weight loss ≥5% group (*P *=* *0.032; Figure 4). The MST of the groups with weight loss <5% and weight loss ≥5% were 23.1 months (95% CI, 19.6–26.6 months) and 14.4 months (95% CI, 10.7–18.1 months), respectively. Multivariate analysis revealed that both a BMI <23 and weight loss ≥5% were independent risk factors for the prognosis of HCC patients who received post-TACE treatment. The other independent factors significantly associated with the OS are summarized in Table 4.

**Table 4.** Univariate and multivariate analysis of factors related to survival in HCC patients who received further treatment after transcatheter arterial chemoembolization

Variable	Univariate analysis *P*-value	Multivariate analysis		
		Hazard ratio	95% CI	*P*-value
Age (≤45 vs >45 years)	0.242	0.932	0.761–1.154	0.542
Sex (female vs male)	0.802	0.994	0.664–1.320	0.706
PT (≤13.5 vs >13.5 s)	0.095	1.035	0.749–1.429	0.836
APTT (≤34 vs >34 s)	0.073	1.081	0.720–1.623	0.706
AST (≤40 vs >40 U/L)	<0.001	1.392	1.101–1.760	*0.006*
ALB (≤40 vs >40 U/L)	0.024	0.887	0.726–1.084	0.242
TB (≤20.5 vs >20.5 μmol/L)	0.038	1.094	0.854–1.402	0.477
AFP (≤25 vs >25 ng/mL)	0.002	1.268	1.018–1.580	*0.034*
BCLC_stage (B vs C)	<0.001	1.685	1.354–2.096	*<0.001*
BMI (<23 vs ≥23 kg/m^2^)	0.005	0.747	0.613–0.911	*0.004*
CWL (<5% vs ≥5%)	0.032	1.339	1.044–1.719	*0.022*

CI, confidence interval; CWL, critical weight loss. Other abbreviations as in Table 1.

## Discussion

In the present study, we found that overweightness was an independent positive prognostic factor for the OS of patients with HCC undergoing TACE as initial treatment. Overweight patients had a significantly longer OS and a higher proportion requiring post-TACE treatment than non-overweight patients. Among the patients who received further treatment, both the overweight and CWL subgroups showed a significant association with the OS.

Obesity can lead to numerous neuroendocrine and immunological changes and contribute to the development of hepatic steatosis, fibrosis, and ultimately HCC [25–27]. However, the relationship between overweightness and the prognosis of HCC in patients remains controversial. In the current study, Cox multivariate analysis demonstrated that overweightness was an independent protective factor for improved OS in HCC patients who initially received TACE treatment. Consistently with our results, the findings of previous studies have demonstrated that overweightness or obesity is as an independent protective prognostic factor for improved tumor-specific survival in patients with other tumor types; this is despite the fact that obesity is a risk factor for the increased incidence of various cancers [28–30]. This phenomenon is referred to as the ‘obesity paradox’ in cancer [31]. Studies have revealed that non-overweight patients have a worse nutritional and physiologic reserve than overweight patients [5, 30]. Non-overweight patients were more susceptible to malnutrition than overweight patients, and studies have shown that malnourished patients experience more accidental treatment interruptions and have shorter OS [32]. Therefore, overweight patients might tolerate therapy better than non-overweight patients, which may ensure the patient’s compliance until the required therapy is completed. In this study, we also found that the proportion of overweight patients receiving subsequent treatment after initial TACE was higher than that of non-overweight patients (63.5% vs 52.8%, *P < *0.001) (Table 1). Therefore, the outcomes of treatment in such patients may be better than that of their non-overweight counterparts. Moreover, overweight patients are thought to have higher economic and educational status in some areas of China [30]. For these reasons, it may be reasonable to assume that overweight patients may be more likely to be able to afford and continue subsequent therapies than non-overweight patients. Additionally, weight is one of the most important factors for assessing malnutrition [33, 34]. Our analysis revealed that the factor of absence of CWL together with younger age (≤45 years) and higher BMI (≥23 kg/m^2^) was an independent favorable factor for the maintenance/improvement of nutritional status (Supplementary Table 1). Malnutrition might contribute to immune suppression. Mello and colleagues [35] suggested that protein malnutrition leads to increased blood levels of interleukin-10, which is regarded as an anti-inflammatory (tumorigenic) cytokine and causes tumor-specific T-cell immunity suppression and promotes cancer growth [36–38]. Therefore, patients with weight maintenance/overweightness are more likely to improve malnutrition and reduce immune suppression.

A recent meta-analysis study, which included 14 retrospective studies, showed that BMI is not an independent prognostic factor for the evaluation of prognosis, including the disease-free survival and OS of HCC patients who have undergone resection [39]. The discrepancy between the results of this meta-analysis and the current study may be attributed to several factors. Generally, the patients who are recommended to undergo surgical resection are at a relatively early stage of the disease, whereas the patients included in the current study who underwent TACE as initial treatment, rather than hepatectomy, had advanced tumor stages. Although the data on the general performance status were not described in these two studies, it may be reasonable to infer that the performance status of patients with early-stage disease who undergo resection would be better than that of patients with advanced stages of the disease. Third, no subsequent treatment is required after radical resection, while subsequent treatment is more common in patients receiving palliative TACE. Therefore, the tolerance of subsequent anticancer treatment may be more important in patients initially treated with TACE, with overweight patients possibly being more tolerant to subsequent treatments than non-overweight patients. However, the precise reason for the differences in the outcomes between surgical treatment and TACE treatment in HCC patients remains to be determined.

Stratified analysis revealed that the OS was longer in overweight patients than in non-overweight patients among those with BCLC B-stage disease. Similarly, among patients with BCLC C-stage diseases, the OS in the overweight sub-category tended to be longer than that in the non-overweight sub-category, although the difference was not significant. The MST in patients with BCLC stage-C disease was 8.0 months (95% CI, 7.0–9.0 months), which was significantly shorter than that in patients with BCLC stage B (16.8 months, 95% CI, 14.9–18.7 months). We believe that the relatively short OS and the small number of patients with BCLC C-stage disease were the main reasons for the lack of a significant survival difference between the overweight and non-overweight patients.

In another stratified analysis, the MST in patients who received post-TACE treatment was 20.6 months (95% CI, 18.0–23.2 months), which was significantly longer than that in patients without subsequent treatment (6.6 months, 95% CI, 5.9–7.3 months). Among patients who did receive post-TACE treatment, the OS was longer in the overweight subgroup than in the non-overweight subgroup. However, no survival benefit of overweightness was found for patients who did not receive any treatment after TACE. As discussed above, overweight patients might tolerate therapy better and may receive adequate therapy; therefore, such patients may have better outcomes. Conversely, the effect of overweightness on the prognosis of patients who did not receive any treatment after TACE was not apparent due to the short OS.

Another independent risk factor for HCC patients receiving post-TACE treatment was CWL. The HR of death among patients with CWL was 1.339 (95% CI, 1.044–1.719; *P *=* *0.022). Consistently with our results for HCC patients, findings of previous studies that investigated other tumor types have also shown the prognostic value of weight loss. For example, Lu and colleagues [15] suggested that weight loss was independently associated with poor survival in patients with advanced esophageal squamous-cell carcinoma. Similarly, Brookman-May and colleagues [16] demonstrated that weight loss predicted the poor outcome of renal-cell carcinoma patients with a BMI of <30 kg/m^2^. Furthermore, Shen and colleagues [40] reported that excessive weight loss had a significant impact on the survival of patients with nasopharyngeal carcinoma. This negative influence of weight loss on cancer survival may be attributed to several factors. Weight loss has been reported to be associated with chemotherapy [14]. Although chemotherapy improves the prognosis in terms of tumor control, aggressive chemotherapy can lead to severe acute toxicities, which increase the patient’s discomfort and difficulty with food intake. Insufficient food intake then impairs the immune system, which further promotes cancer progression [14, 41]. Moreover, patients without CWL might have a very tolerant nutritional status and may therefore have a better prognosis [42]. In fact, weight loss has been regarded as one of the hallmarks of cachexia, and cachexia was defined as weight loss greater than 5% [43, 44]. Cancer cachexia is observed in 80% of patients with advanced-stage diseases and is one of the crucial causes of cancer-related mortality [45, 46]. Previous studies demonstrated that nutritional intervention to weight gain improves the OS of HCC patients [47–49]. In this present study, we also found that HCC patients with weight gain >5% after TACE treatment have longer OS than those with weight gain ≤5% (including weight loss, weight maintenance, and weight gain ≤5%) (Supplementary Figure 3 and Supplementary Table 2). However, the exact mechanism by which nutritional intervention (aimed to fuel weight gain) improves the OS of HCC patients is unclear. One possible explanation is that hypoalbuminemia is an independent unfavorable factor of OS and/or recurrence rate for HCC [50]. Nutritional support for HCC patients significantly increases the serum albumin level, reduces the incidence of ascites or peripheral edema, and contributes to the improvement in malnutrition [49, 51, 52]. Second, glutamine, an amino acid, is used as a source of energy and nitrogen by rapidly dividing cells and cancer patients require more of this amino acid. Deficiency of glutamine may lead to changes in their immune status, the integrity of the intestinal mucous membrane, and protein-energy metabolism, contributing to cancer cachexia [53, 54]. Nutritional intervention with glutamine improves the metabolism and the clinical condition of the patient without increasing cancer growth and appears to support the efficacy of chemoradiotherapy treatment while reducing the toxicity of the tissues. Third, the patients receiving nutritional intervention with arginine and branched amino acid had a reduced occurrence rate of complications and length of hospital stay, and improved quality of life and treatment tolerance [49, 55, 56].

In the current study, the HGB level (141.59 ± 18.07) in the overweight group was significantly higher than that (137.15 ± 20.54) in the non-overweight group, which is consistent with previous studies [57–61]. In a cross-sectional study, the associations between hemoglobin and alcohol consumption or physical activity have been reported [58]. However, the exact mechanism by which overweight people have higher HGB than non-overweight people is not clear.

Our study has some limitations. First, all of the included patients were from a single center, and therefore it is necessary to determine how the results of this study can be extrapolated to other populations. Second, the post-TACE weight measurement of patients who did not receive subsequent treatment was not obtained. Therefore, the impact of weight loss on HCC patients who were treated with only a single cycle of TACE remains unclear. Third, and most importantly, the exact underlying mechanisms of the effects of overweightness and weight loss on the prognosis of HCC patients are unknown. Therefore, immunological and biological investigations are imperative to elucidate the underlying mechanisms.

In summary, our results demonstrate that overweightness is associated with longer OS of HCC patients undergoing TACE as initial treatment. Moreover, CWL was identified as a negative prognostic factor for HCC patients receiving further treatment after TACE. These data suggest that the nutritional status of HCC patients who are initially treated with TACE is significantly related to their survival, and therefore proper nutritional guidance should be provided for such patients.

## Authors’ contributions

Z.X.C. and X.M.L. conceived of and designed the project. Z.X.C., Z.W.J., X.W.W., J.C.W., and J.Y.P. collected the data. Z.X.C., Z.W.J., and X.M.L. analysed and interpreted the data. Z.X.C., Z.W.J., X.W.W., J.C.W., J.Y.P., C.Y.H., and X.M.L. drafted the manuscript. All authors read and approved the final manuscript.

## Funding

This work was partly supported by a project grant from the National Natural Science Foundation of China [No. 81773057].

## Supplementary Material

goz040_Supplementary_Data
