# Supplementary material for: Impact of overweightness and critical weight loss on overall survival in patients with hepatocellular carcinoma initially treated with chemoembolization
Source: Gastroenterol Rep (Oxf). 2019 Aug 28;8(2):125–33. doi: 10.1093/gastro/goz040 (PMC7136712; doi:10.1093/gastro/goz040)
Supplement: goz040_Supplementary_Data [file goz040_supplementary_data.zip › goz040-Suppl_Data/2019-072 supplementary figure legends and Tables 1-2.docx]

**Figure S1** Kaplan–Meier curves of overall survival (OS) for hepatocellular carcinoma (HCC) patients by body mass index (BMI) level. A. For the 447 overweight patients, there was no significant difference between the at-risk (23–24.9 kg/m^2^) and obese (≥25 kg/m^2^) subcategories. B. For the 723 non-overweight patients, no significant difference was noted between those underweight (<18.5 kg/m^2^) and normoweight (18.5-22.9 kg/m^2^). TACE, transarterial chemoembolization; CI, confidence interval.

**Figure S2** Kaplan–Meier curves of overall survival (OS) for 1170 hepatocellular carcinoma (HCC) patients with or without further treatment after initial treatment with transarterial chemoembolization (TACE). Patients with subsequent treatment had significantly higher OS than patients without subsequent treatment. CI, confidence interval.

**Figure S3** Kaplan–Meier curves of overall survival (OS) for 654 hepatocellular carcinoma (HCC) patients (for patients without post-TACE treatment, the post-TACE weight measurements were not available) receiving further treatment after transarterial chemoembolization (TACE), classified by weight gain status. The OS of the group with weight gain of >5% was significantly greater than that of the group with weight gain of ≤5%. TACE, transarterial chemoembolization; CI, confidence interval.

**Table S1 Univariate and multivariate analysis of factors related to maintenance/improvement of nutritional status in all HCC patients initially treated with TACE**

| **Characteristic** | **Univariate analysis**  ***P* value** | **OR** | **95%CI** | **Multivariate analysis**  ***P* value** |
| --- | --- | --- | --- | --- |
| Age (≤45: >45 years) | 0.004 | 2.119 | 1.239-3.623 | *0.006* |
| Gender (female: male) | 0.210 |  |  |  |
| Viral infection (yes: no) | 0.193 |  |  |  |
| BMI (<23: ≥23 kg m-2) | 0.032 | 0.572 | 0.357-0.915 | *0.020* |
| CWL (<5: ≥5 %) | 0.012 | 2.079 | 1.239-3.484 | *0.006* |

BMI, body mass index; CWL, critical weight loss; OR, odds ratio; CI, confidence interval.

**Table S2 Univariate and multivariate analysis of factors related to survival in HCC patients who received further treatment after transcatheter arterial chemoembolization**

| Variable | Univariate analysis  *P* value | Multivariate analysis | | | | |
| --- | --- | --- | --- | --- | --- | --- |
|  |  | Hazard ratio | | 95% CI | | *P* value |
| Age (≤45: >45 years) | 0.242 | 0.925 | 0.750-1.140 | | 0.465 | |
| Gender (male: female) | 0.802 | 0.957 | 0.680-1.347 | | 0.802 | |
| PT (≤13.5: >13.5 s) | 0.095 | 1.073 | 0.776-1.484 | | 0.671 | |
| APTT (≤34: >34 s) | 0.073 | 1.112 | 0.741-1.667 | | 0.608 | |
| AST (≤40: >40 U/L) | <0.001 | 1.423 | 1.127-1.798 | | *0.003* | |
| ALB (≤40: >40 U/L) | 0.024 | 0.889 | 0.727-1.086 | | 0.249 | |
| TB (≤20.5: >20.5 μmol/L) | 0.038 | 1.087 | 0.849-1.392 | | 0.508 | |
| AFP (≤25: >25 ng/mL) | 0.002 | 1.267 | 1.018- 1.578 | | *0.034* | |
| BCLC_Stage (B: C Stage) | <0.001 | 1.699 | 1.366-2.112 | | *<0.001* | |
| BMI (<23: ≥23 kg m^-2^) | 0.005 | 0.748 | 0.615-0.910 | | *0.004* | |
| Weight gain (≤5: >5 %) | 0.043 | 0.616 | 0.428-0.885 | | *0.009* | |

Abbreviations as in Table 1.
