# Supplementary figures and images for: Impact of overweightness and critical weight loss on overall survival in patients with hepatocellular carcinoma initially treated with chemoembolization
Source: Gastroenterol Rep (Oxf). 2019 Aug 28;8(2):125–33. doi: 10.1093/gastro/goz040 (PMC7136712; doi:10.1093/gastro/goz040)

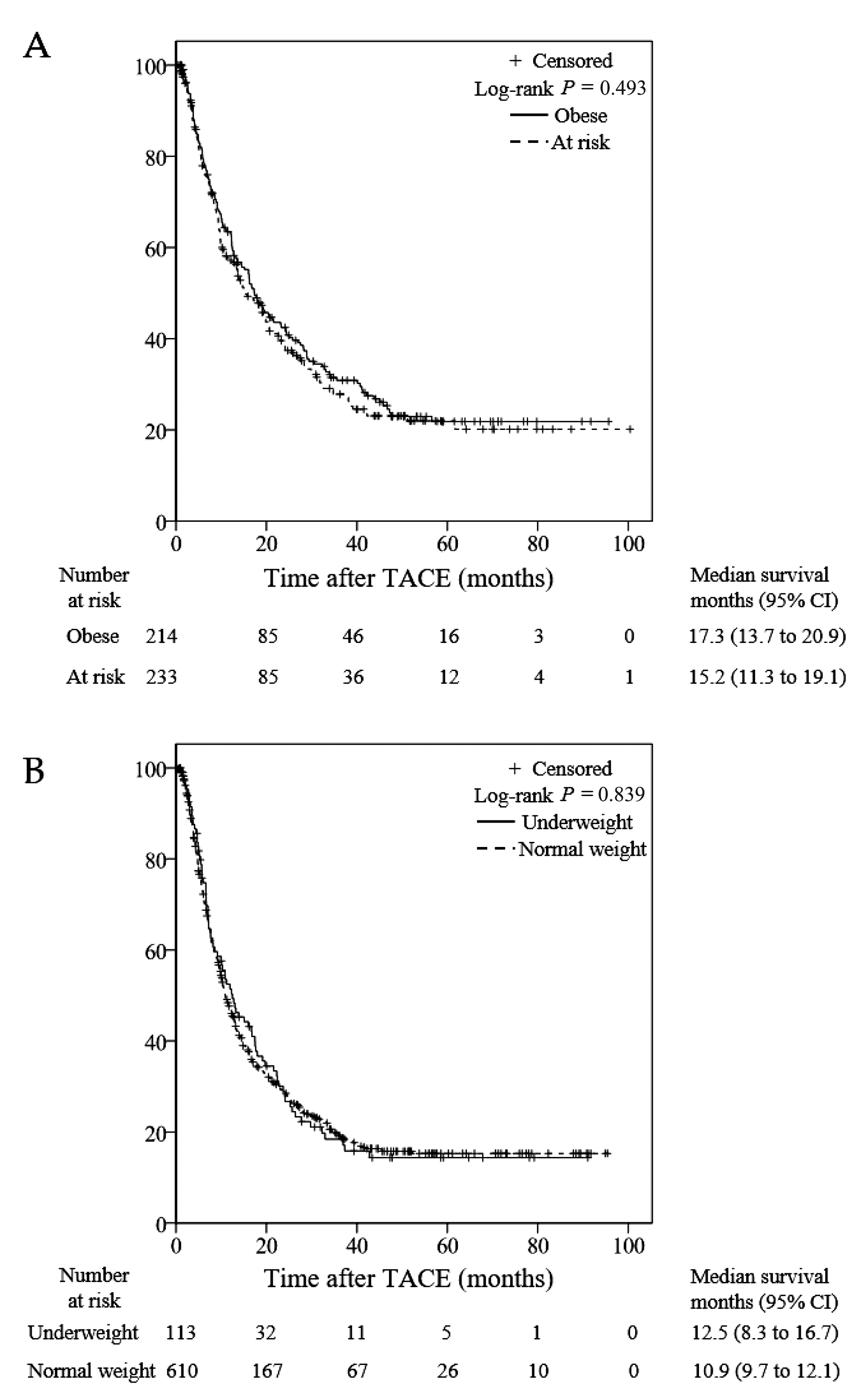

Supplement: goz040_Supplementary_Data [file goz040_supplementary_data.zip › goz040-Suppl_Data/2019-072 Figure S1.tif]

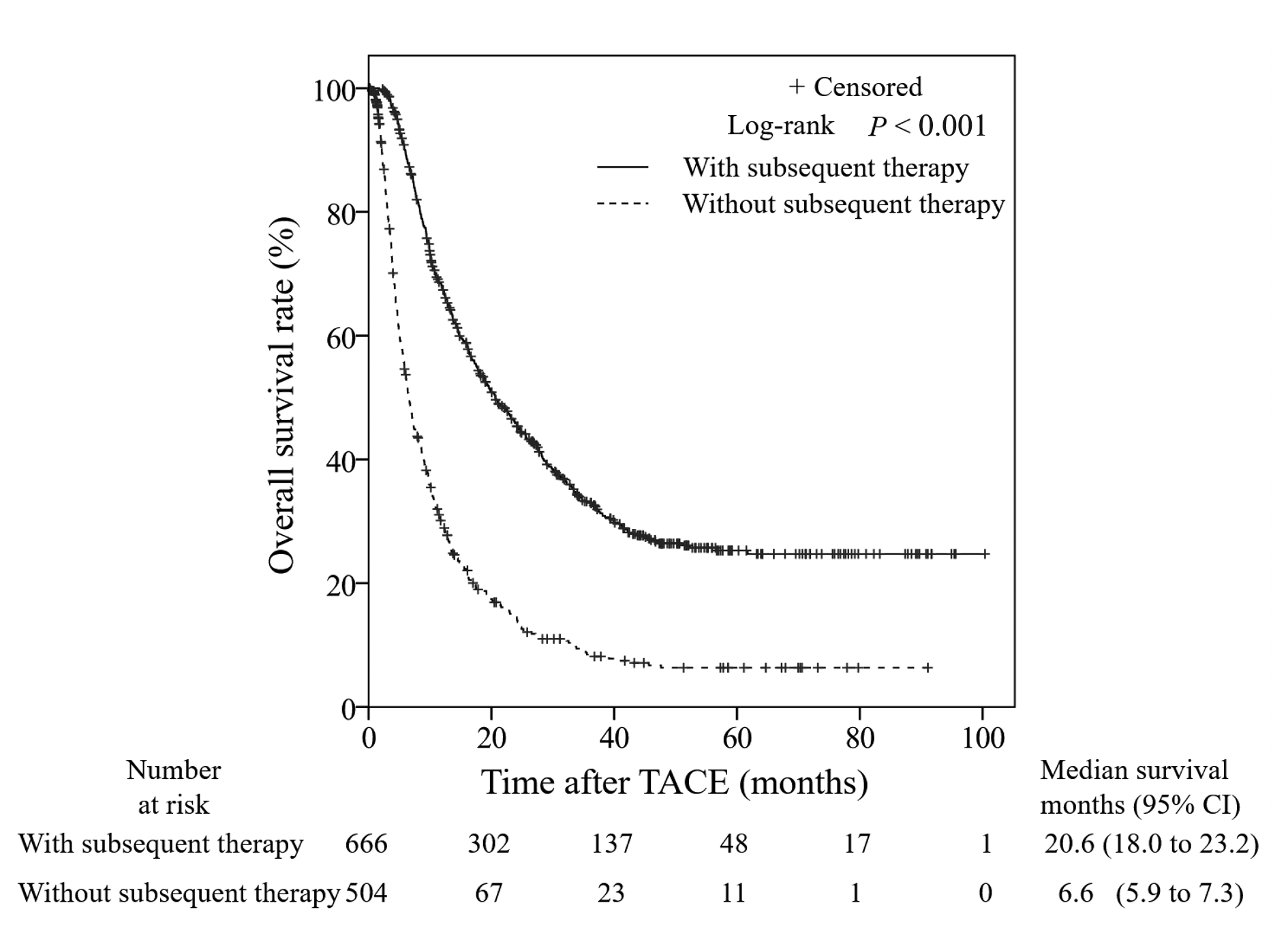

Supplement: goz040_Supplementary_Data [file goz040_supplementary_data.zip › goz040-Suppl_Data/2019-072 Figure S2.tif]

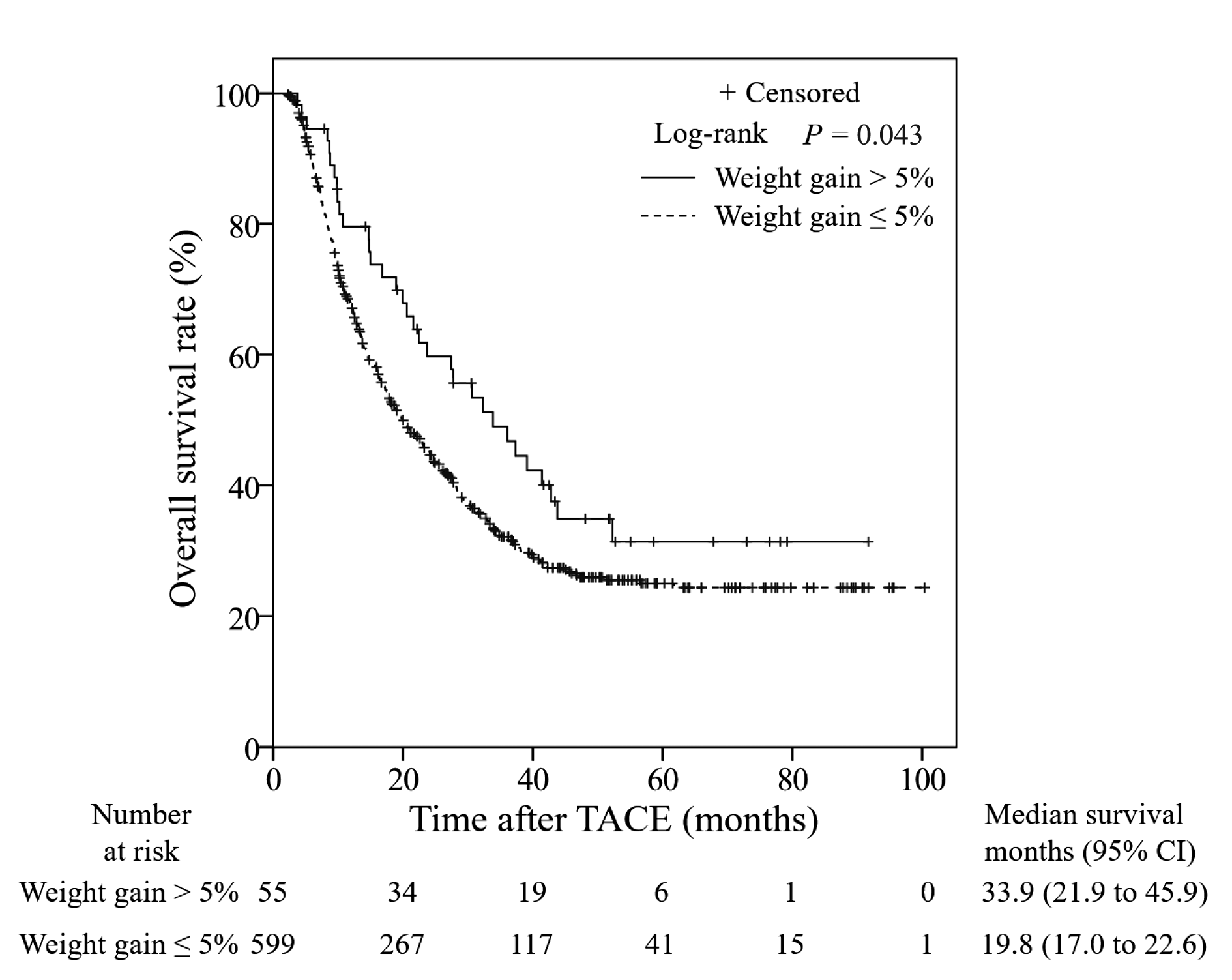

Supplement: goz040_Supplementary_Data [file goz040_supplementary_data.zip › goz040-Suppl_Data/2019-072 Figure S3.tif]
